# Nanopore-induced host–guest charge transfer phenomena in a metal–organic framework†
†Electronic supplementary information (ESI) available: Crystallographic data, structural details, and additional experimental results, such as cyclic voltammetry, conductivity measurements, SEM-EDX results and PXRD patterns, are included. CCDC 1812408 and 1812409. For ESI and crystallographic data in CIF or other electronic format see DOI: 10.1039/c7sc05390h


**DOI:** 10.1039/c7sc05390h

**Published:** 2018-02-15

**Authors:** S. Yamamoto, J. Pirillo, Y. Hijikata, Z. Zhang, K. Awaga

**Affiliations:** a Department of Chemistry , Nagoya University , Furo-cho, Chikusa-ku , Nagoya 464-8602 , Japan . Email: zhang.zhongyue@i.mbox.nagoya-u.ac.jp ; Email: awaga@mbox.chem.nagoya-u.ac.jp; b Institute of Transformative Bio-Molecules (WPI-ITbM) , Nagoya University , Furo-cho, Chikusa-ku , Nagoya 464-8602 , Japan; c Integrated Research Consortium on Chemical Sciences (IRCCS) , Nagoya University , Furo-cho, Chikusa-ku , Nagoya 464-8602 , Japan

## Abstract

A spontaneous entrapment of electron-donating small guest molecules, including tetrathiafulvalene (TTF) and *N*,*N*,*N*′,*N*′-tetramethyl-1,3-propanediamine (TMPDA), was realized in a structurally flexible metal–organic framework, {Mn_7_(2,7-AQDC)_6_(2,6-AQDC)(DMA)_6_}_∞_ (AQDC = anthraquinone dicarboxylates, DMA = *N*,*N*-dimethylacetamide), with electron-accepting anthraquinone groups, generating two MOF guest charge transfer complexes: {Mn_7_(2,7-AQDC)_6_(2,6-AQDC)(DMA)_6_(TTF)_5_} and {Mn_7_(2,7-AQDC)_6_(2,6-AQDC)(DMA)_4_(H_2_O)_2_(TMPDA)_7_}. Using a mild impregnation procedure, single crystals of the target complexes were obtained *via* a crystal-to-crystal conversion, and the crystals were suitable for structural analysis. Single crystal X-ray analysis demonstrated the different arrangements of these intercalated donor molecules: some donor molecules interacted with the anthraquinone groups and formed infinite D–A–A–D stacks, some appeared beside the anthraquinone groups but only formed donor–acceptor pairs, and the remainder of the molecules simply filled the space. The charge transfer between the guests and the framework was spectroscopically confirmed, and the radical densities on the organic species were estimated using magnetic susceptibility measurements. Compared with a solid-state mixture of anthraquinone and donor molecules, the evenly distributed donor molecules in the micropores of the MOF resulted in a “solid solution” state and significantly promoted the degree of charge transfer between donors and acceptors. Such an encapsulation process may be adopted as a new strategy for post-modification of the electronic and magnetic properties of MOFs, as well as for generating new semiconducting charge-transfer complexes.

## Abstract 2

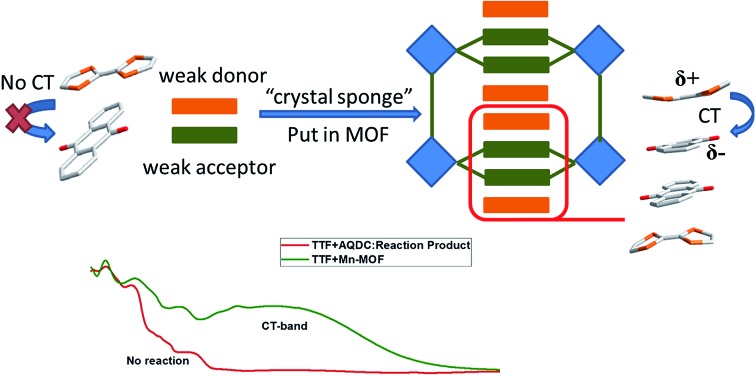
Using the “crystal sponge” approach, weak organic electron donor molecules were impregnated and evenly distributed in a crystal of a metal–organic framework (MOF), with the self-assembly of the donor–acceptor pairs with electron acceptor ligands. The nanopores of the MOF confined them and induced a charge transfer phenomenon, which would not occur between donor and acceptor molecules in a bulk scale.

## Introduction

Metal–Organic Frameworks (MOFs) are a series of polymeric coordination compounds with intrinsic porosity and crystallinity.^1^–^3^ As a result of these intrinsic properties, small guest molecules can be entrapped inside the internal void space of MOFs without significant perturbation of the structure. This property has led to various applications of MOFs, such as for gas adsorption^4^,^5^ and separation,^6^,^7^ molecule sensing,^8^,^9^ catalysis,^10^–^12^ hazardous waste treatment^13^,^14^ and photoluminescence.^15^,^16^ Indeed, most of the studies on MOFs have concluded that the interactions between guest species and host frameworks have played a crucial role in the aforementioned applications. With a proper design strategy, interactive spots such as open coordination sites^17^,^18^ and organic functional groups^19^,^20^ can be installed on the metal-cluster junctions and ligands, respectively. As a result, specific guest molecules will be bonded to these spots and entrapped in the scaffold, leading to selective adsorption,^21^ purification^22^ and catalysis.^23^ On the other hand, the conductive properties of MOFs have rarely been discussed,^24^–^26^ let alone the possibility of manipulating them *via* the insertion of certain guest molecules. A few elegant examples have been reported on this topic: Allendorf *et al.* reported the synthesis of TCNQ@HKUST-1 (TCNQ = 7,7,8,8-tetracyanoquinodimethane, HKUST-1 = {Cu_3_(benzene-1,2,4-tricarboxylic acid)_2_}), and the conductivity of HKUST-1 was improved by six orders of magnitude with the infiltration of TCNQ.^27^ Dincă *et al.* illustrated VOC (volatile organic compound) detection with thin-film devices made of two-dimensional conductive MOFs.^28^ Zuo *et al.* demonstrated the iodine oxidation of TTF (tetrathiafulvalene)-based MOFs, and found that the oxidation modulated the spin-crossover property of the MOF.^29^,^30^ Although these reports provide evidence that the impregnated guest molecules could modulate the electronic and magnetic properties of MOF materials, unfortunately, neither of the studies offered a detailed explanation for the mechanism of such modulation. Neither the oxidation states nor the spin density of frameworks and inserted guests has been determined with solid evidence. Indeed, the main obstacle here is the lack of fully resolved crystal structures. Obtaining high quality single crystals of guest impregnated MOFs is tremendously difficult, especially when a strong electrostatic interaction is expected between the guest and the framework.

To understand how guest molecules have tuned the electron transfer ability of MOFs, an important lesson should be learned from the studies of charge transfer molecular conductors such as TTF–TCNQ^31^ and the complexes based on their derivatives. Because they are charge transfer salts composed of pairs of organic donors and acceptors, these materials exhibited exceptionally high electrical conductivity for molecular materials. Even metallic conductivity was observed in many of the charge transfer complexes.^32^,^33^ Owing to the high quality single crystals that have been prepared through the self-assembly of donor and acceptor molecules in solution, the electron transfer characteristics and energy band structures of these materials could be computationally simulated using their crystal structures. Therefore, the crystal structures of MOF–guest complexes are a prerequisite for understanding their conductive behaviours, and the resolution must be adequate for resolving all numerical details of the structures.

**Scheme 1:**
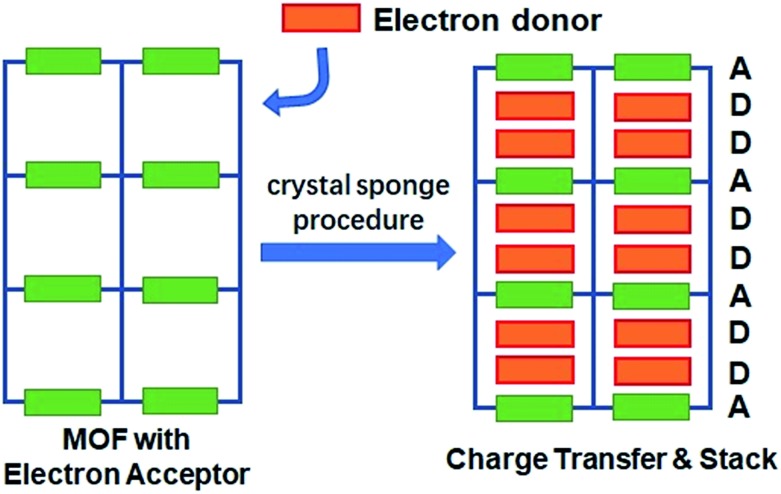
The conceptual scheme for preparing MOF guest D–A charge transfer complexes *via* the “crystal sponge” approach.

Fujita *et al.* have provided a straightforward yet universal strategy for this goal—namely, the “crystal sponge” strategy.^34^,^35^ In this approach, the crystals of host porous materials are placed in a solution of guest molecules, and the solvent is slowly evaporated, leaving the internal space of the cages fully occupied by the guest species. Using this method, high quality crystals suitable for X-ray analysis could be realized, and thus the structure of the guest molecules could be revealed in a porous coordination cage *via* this mild guest inhalation process. Such a gentle self-assembly process may not only lead to the impregnation of structurally resolvable guest molecules in a confined space, but may also provide an environment for maximizing the interaction between donor–acceptor pairs and optimizing their packing motifs (Scheme 1). Herein, by applying this strategy to a structurally flexible, redox-active MOF, {Mn_7_(2,7-AQDC)_6_(2,6-AQDC)(DMA)_6_} (Mn-MOF), that was previously reported by our group,^36^ we achieved two MOF guest charge transfer complexes: {Mn_7_(2,7-AQDC)_6_(2,6-AQDC)(DMA)_6_(TTF)_5_} (Mn-MOF 5TTF, **1**) and {Mn_7_(2,7-AQDC)_6_(2,6-AQDC)(DMA)_4_(H_2_O)_2_(TMPDA)_7_} (Mn-MOF 7TMPDA, **2**, TMPDA = *N*,*N*,*N*′,*N*′-tetramethyl-1,4-phenylenediamine). They were composed of organic donor guests and an Mn-MOF with electron-accepting anthraquinone (AQ) groups. The crystal structures were successfully acquired through a spontaneous crystal-to-crystal transformation using the “crystal sponge” method, and the charge transfer between the MOF scaffold and organic guest donor molecules was evidenced by a series of physical property measurements.

## Experimental methods

### Preparation of the materials

The preparation of a Mn-MOF was first described by our group.^36^ In the same report, we also described the “breathing” properties of the framework upon the removal and re-introduction of solvent molecules. Since multiple crystal-to-crystal transformation steps during the solvent exchange may create cracks on the crystals, the as-prepared Mn-MOF crystals were collected from the product of the solvothermal reactions, washed with hot DMF and used without solvent exchange and activation. Five milligrams of these crystals were placed in a small vial, followed by the addition of 5 mL of a dichloromethane solution with ∼10 mg TTF or TMPDA guest molecules. The vial was capped and the cap was penetrated with a needle. The needled vial was left in the fume hood, allowing slow evaporation of solvent. After 2–3 days, the vial was dried, and the leftover solid materials were a mixture of Mn-MOF guest crystals and excess guest molecules. The vial was then placed in a larger flask, which was vacuumed and heated to 140 °C. The excess free organic molecules were removed by sublimation, leaving phase-pure Mn-MOF guest crystals.

### X-ray diffraction analysis: single crystal and powder

X-ray diffraction analysis (XRD) was performed on the single crystals of **1** and **2**. Single crystal XRD data collection was first carried out on these crystals using a Rigaku RA-micro7 Mercury CCD diffractometer equipped with a Mo Kα light source (*λ* = 0.71073 Å) at –150 °C. Two hemispheres were scanned for each crystal. The frames were integrated with Rigaku Crystal Clear 2.0, and the solving and refinement of structures were completed with Rigaku Crystal Structure 2.0. Based on the crystallographic results from single crystal XRD, we concluded that the data quality of **2** was not adequate for scientific publication. To overcome this problem, synchrotron radiation diffraction was also performed on a crystal of **2** at the Aichi Synchrotron Radiation Center, with beamline BL2S1 (*λ* = 0.75000 Å). The instrument conditions of the beamline were formerly reported,^37^ and a full spherical scan was performed on the crystal since the instrument was equipped with a single-axis goniometer. The diffraction data were also attained at –150 °C, and the data reduction, solution and refinement were performed with Rigaku Crystal Structure 2.0. All non-H atoms were anisotropically refined, and the PLATON/SQUEEZE function was used to remove the electron density from the disorder of some methyl groups on DMA and TMPDA molecules in **2**. The powder X-ray diffraction patterns were collected on a Rigaku MultiFlex X-ray diffractometer, and the crystals of the as-prepared Mn-MOF guest complexes were roughly ground by sandwiching them between two glass slides to create a crystalline powder. A simulated powder diffraction pattern was generated from the crystal structure using Diamond 3.0 crystallographic imaging software for comparison.

### Physical measurements

A series of physical properties were characterized to investigate the existence of charge transfer phenomena between the host scaffold and the encapsulated guest molecules. The as-prepared Mn-MOF, organic donor molecules, and Mn-MOF guest crystals were analysed by solid-state UV-vis-NIR diffuse reflectance spectroscopy using a JASCO V-570 UV-Vis-NIR spectrophotometer. Due to the limited amount of Mn-MOF guest crystals, the diffuse reflectance measurements were performed by mixing the crystals with BaSO_4_ powder with a weight ratio of 1 : 10. The spectrum was taken within a wavelength range of 200–1400 nm. To verify the effect of micropores on the charge transfer degree between the donor molecules and functional groups on the MOF, we also performed a reaction between the donor molecules and anthraquinone-containing ligands by dissolving them in hot DMF (*N*,*N*-dimethylformamide) with a 1 : 1 molar ratio. The DMF solution was kept at 120 °C for 1 hour and the solid-state products were collected by evacuating all the DMF solvent. These products were also examined by solid-state UV-vis diffuse reflectance spectroscopy under the same conditions.

The charge transfer degree between the donor molecules and anthraquinone groups was evaluated by temperature variable magnetization measurements, which were performed with a superconducting quantum interference device (SQUID) Quantum Design MPMS system. DC measurement was performed with a magnetic field of 1000 Oe, and the temperature range was 2–300 K. The *χ*–*T* plot data sets were fitted using the Curie–Weiss law, and the Curie constants were used to calculate the magnetic contribution of the organic radicals. To confirm the presence of organic radicals and exclude the contribution of paramagnetic impurities, temperature variable electron paramagnetic resonance (EPR) measurements were also performed on Mn-MOF and Mn-MOF guest crystals. A JEOL JES-FA200 continuous wave X-band EPR instrument was used to complete this task.

## Results and discussion

### Structural perspectives

**Fig. 1:**
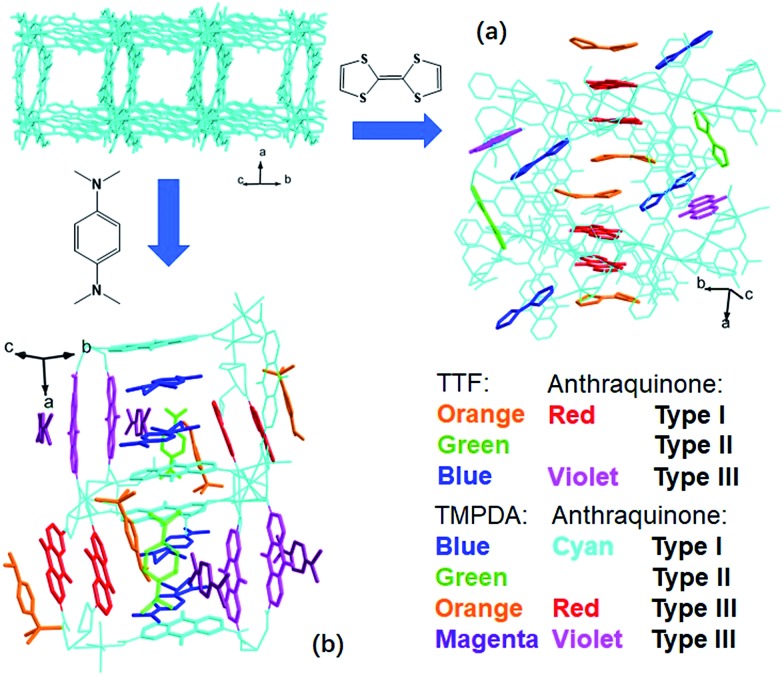
Incorporation of organic electron donors into the nanochannel of Mn-MOF using the crystal sponge method, forming Mn-MOF 5TTF (a) and Mn-MOF 7TMPDA (b). Type I donor molecules formed infinite D–A–A–D stacks with AQ groups on the skeleton, Type II donor molecules just filled the space, and Type III donor molecules formed D–A pairs or D–A–A–D units with AQ groups, but they were not connected into columns.

The host framework, Mn-MOF, is a flexible MOF with anthraquinone functional groups and was previously prepared by our group.^36^ Organic donors, including TTF and TMPDA, were inserted into the framework by a typical “crystal sponge” procedure: immersing pristine MOF crystals in a CH_2_Cl_2_ solution of these donors and evaporating the solvent. This procedure yielded MOF guest complexes. Fortunately, high quality single crystals could be obtained for both charge transfer complexes, which makes it easy to inspect the structural features of the donor–acceptor pairs in the Mn-MOF guest crystals. After the insertion of the donor molecules, the skeleton of Mn-MOF was not significantly perturbed; however, astonishingly complicated arrangements of organic donor molecules were observed in the structures of both **1** and **2** (Fig. 1). In general, three types of donor molecule were observed in both cases: two Type I donor molecules appeared above and below a pair of anthraquinone groups, forming a short D–A–A–D unit, and infinitely repeated units were connected into a …D–A–A–D···D–A–A–D… column. In Fig. 1, this D–A–A–D column is highlighted with orange (for TTF) and red (for anthraquinone) for Mn-MOF TTF, while the pack is highlighted in blue (for TMPDA) and cyan (for anthraquinone) for Mn-MOF TMPDA. Type II donor molecules were located with no adjacent anthraquinone acceptors around them, and merely played a space-filling role. In Fig. 1, these donor molecules are highlighted in green in both structures. Type III donor molecules were also positioned beside the anthraquinone groups, but no column could be formed in the crystal. TTF molecules were accompanied by 2,6-AQDC, isolated D–A pairs were formed (highlighted in blue and violet), and four TMPDA molecules sandwiched two pairs of 2,7-AQDC, resulting in two crystallographically independent D–A–A–D units, but these units were far away from each other and no infinite stacks were formed. The TMPDA molecules in these units are individually coloured magenta and orange, while their counterpart anthraquinone groups are shown in red and violet. In both structures, columns of infinitely stacked donors and acceptors were achieved, which provides a potential pathway for electron transfer and may convert the original insulating MOF into a semiconducting material.

According to the previous research on charge transfer complexes,^38^,^39^ the valency of organic molecules, as well as the electron transporting properties of the materials, could be predicted by analysing the details of their structures, such as bond lengths, bond angles, separation distances and the packing motifs of donor and acceptor species. Therefore, it is critical to carefully examine these factors for donor molecules, anthraquinones and D–A–A–D columns in these structures.

**Fig. 2:**
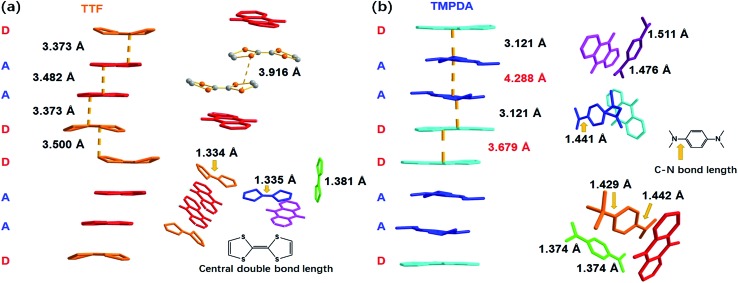
The crystallographic details of compound **1** (a) and **2** (b), where the central bond lengths of TTF molecules and the lengths of C–N bonds that connect the phenyl rings and amino groups in the TMPDA molecules are displayed. These parameters indicate the oxidation states of donor molecules.

As shown in Fig. 2a, a side view of the TTF-anthraquinone stacks in compound **1** indicated that the TTF molecules in the stacks are slightly curved. Structure factors revealed that the sulphur atoms and carbon atoms of the central C=C double bond are still coplanar, while the five-membered rings are bent along the dithiole line with a torsion angle of ∼30°. Such distortion should be a result of weak attractive intermolecular S···S interactions, as the S···S distance between the adjacent TTF molecules is 3.916 Å.^40^ The separation distances between the molecules in the D–A–A–D columns are 3.373 Å (D–A separation), 3.482 Å (A–A separation) and 3.500 Å (D–D separation). These distances are adequate for electron transfer between the neighbouring molecules, but the inhomogeneous separation distances alternating in a long-short-long pattern hinted at an electrostatic attraction between the positively charged TTF molecules and negatively charged AQ groups, as well as the repulsion between D–D and A–A spacings. Thus, the electron density distribution within the column might be strongly localized due to these electrostatic interactions. The lengths of the central C=C double bonds of TTF molecules were also examined, since these bond lengths are benchmarks for predicting the oxidation states of TTF species. Interestingly, abnormalities were observed in various types of TTF molecule in the structure. Because they are adjacent to an AQ group, the Type I and Type III TTF molecules were expected to possess a positive charge, and the central C=C bond was expected to be elongated by the single bond component from the resonance structure compared to that in the neutral, space-filling Type II TTF molecules. In reality, the C=C bond lengths of Type I and Type III TTF were 1.334 Å and 1.335 Å, respectively, and Type II TTF had a central C=C bond length of 1.383 Å. As a reference, this bond length was 1.337 Å in neutral TTF and 1.369 Å in TTF–TCNQ (*δ*_TTF_ = +0.59).^41^ Obviously, for **1**, it is inappropriate to simply predict the charge transfer degree with bond lengths, and the bond lengths of impregnated TTF are determined by not only the oxidation states, but also the steric effects arising from the limited space in the nanopores in the MOF structure.

A few of the aforementioned structural features were also observed in compound **2** (Fig. 2b). For instance, the separation distances in the D–A–A–D columns also alternated in a long-short-long manner: the D–A separation distance was 3.121 Å, and the D–D and A–A separation distances were 3.679 Å and 4.288 Å, respectively. Compared with that in **1**, the heterocharge separation distance in **2** is shorter and the homocharge separation distances are further, suggesting stronger electrostatic attractions and repulsions within the columns. This phenomenon indicates a larger charge transfer degree and higher charge density on the donor and acceptor species, which is consistent with the fact that the electron-donating ability of TMPDA is stronger than that of TTF (see the cyclic voltammetry results in Fig. S2†). Meanwhile, since former researchers have suggested that the lengths of the C–N bonds that connect the phenyl rings and dimethylamino groups were benchmarks for the oxidation states of TMPDA,^42^ these bond lengths in **2** were also examined and compared with those of the neutral species and charge transfer organic salts of TMPDA. Again, we found that the bond lengths in **2** contradicted the empirical rules in preliminary reports: the C–N bond lengths in neutral TMPDA^0^ were reported to be 1.41–1.43 Å, and the C–N bonds in monovalent TMPDA species were 1.34–1.37 Å. These shortened C–N bond lengths in positively charged TMPDAs could be explained by their quinoid character. But in **2**, the space-filling TMPDA molecule (green, see Fig. 2b) possesses the shortest C–N bond lengths, while the other TMPDA molecules, which are supposed to be oxidized, have much longer C–N bond lengths. This unusual feature may be induced by the different hybridization models of the N atoms. In all the previous reports, the TMPDA molecules were planar and the N atoms adopted sp^2^ hybridization, while in compound **2**, all TMPDAs adjacent to AQ groups were slightly twisted from the planar configuration, and the methyl groups on amino groups were pointing out of the benzene plane (Fig. S4†). This finding illustrated the partial sp^3^ hybridization character of these N atoms, and not surprisingly, the C–N bonds were elongated. Given that the evaporation of solvent was performed in air and that two coordinated DMAs were replaced by water molecules, one reasonable assumption would be that a small proportion of the TMPDA molecules were protonated by the ambient water. Such protonation has been observed in a reaction between TMPDA and 2,4,6-tricyano-1,3,5-triazine under standard laboratory conditions, yielding [HTMPDA^+^][DCTO^–^] (DCTOH = 2,4-dicyano-6-hydroxy-*s*-triazine).^43^ The structural analysis of **1** and **2** showed a self-arranged placement of donor molecules in these MOF–guest complexes. With the “crystal sponge” approach, donor molecules will automatically find acceptors, forming columns that are potentially capable of electron transfer.

### Experimental evidence of charge transfer between MOF and guest molecules

**Fig. 3:**
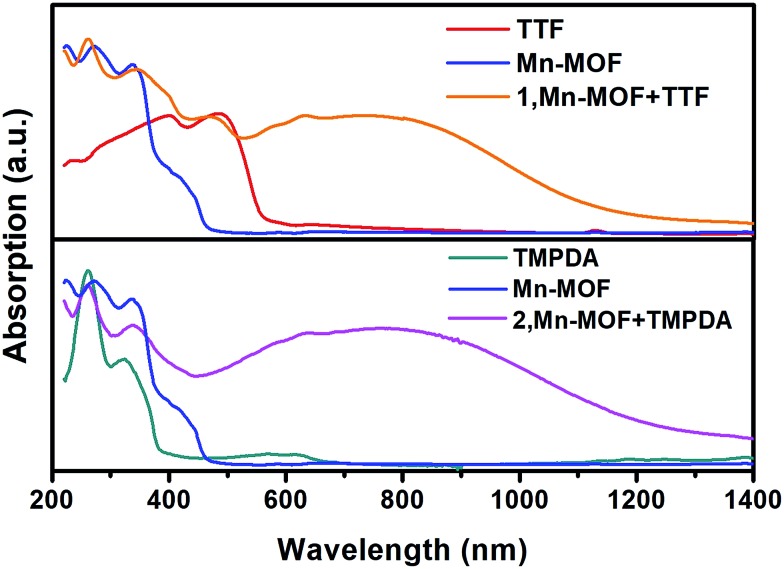
Solid-state UV-vis-NIR diffusive reflectance spectra of the original MOF, donor molecules and MOF–guest complexes. Charge transfer (CT) bands were observed.

Although the crystal structures of **1** and **2** have provided quite a few hints of charge transfer taking place between AQ groups and donor molecules, evidence from physical measurements is still essential to prove the existence of organic radical species. We therefore conducted solid-state diffusive reflectance UV-vis spectroscopy measurements on the original MOF, donor molecules and MOF–guest complexes. By overlaying the absorbance spectrum of the complexes with those of the donor molecules and Mn-MOF, broad new bands were observed for both **1** and **2** at 740 nm and 790 nm, respectively (Fig. 3). These bands were a straightforward illustration of electron transfer between the donor molecules and AQ groups. Since charge transfer will generate organic radical species, the average valencies of TTF and TMPDA were estimated by temperature variable magnetic susceptibility measurements. Mn-MOF, **1** and **2** were characterized with a SQUID magnetometer, and all three compounds exhibited typical Curie–Weiss behaviour (Fig. S9†). For the original Mn-MOF, Curie–Weiss law fitting of data from 50–300 K provided a Curie constant of 24.84 cm^3^ mol^–1^ K, and this value was much lower than the sum of the values of seven isolated high spin Mn(ii) (*n* = 7, *S* = 5/2, *g* = 2.0, *C* = 30.63 cm^3^ mol^–1^ K for the contribution of isolated spins) cations. This small Curie constant could be simply explained by the antiferromagnetic (AF) exchange coupling between the Mn(ii) cations within one secondary building unit (SBU). To simplify the analysis, we decided to use the giant spin approximation^44^ and therefore the magnetic susceptibility of an SBU in Mn-MOF could be simulated as a spin with *S* = 13/2, *g* = 2.04 and *zJ* = –0.177 cm^–1^ (*zJ* = mean intermolecular interaction, see ESI†). The magnetic contribution of organic radicals could be roughly assessed by deducting the “background” Curie constant of this giant spin from the fitted value of **1** and **2**. By fitting the 1/*χ*–*T* data of **1** and **2** to the Curie–Weiss law within a temperature range of 50–300 K, the Curie constants of **1** and **2** were calculated as 24.95 cm^3^ mol^–1^ K and 30.46 cm^3^ mol^–1^ K, respectively. Considering the magnetic contribution from the isolated organic radicals with *S* = 1/2, we estimated the charge of the donor molecules in **1** and **2** to be (TTF_5_)^0.15+^ Mn-MOF^0.15–^ and (TMPDA_7_)^7+^ Mn-MOF^7–^. Although this estimation ignored the potential AF interactions between AQ˙^–^ and the donor˙^+^, as well as the AF interactions between metal clusters and organic radicals, it was still qualitatively shown that the impregnated TTF molecules were almost neutral, and the main character of the TMPDA-AQ charge transfer pairs was ionic.

**Fig. 4:**
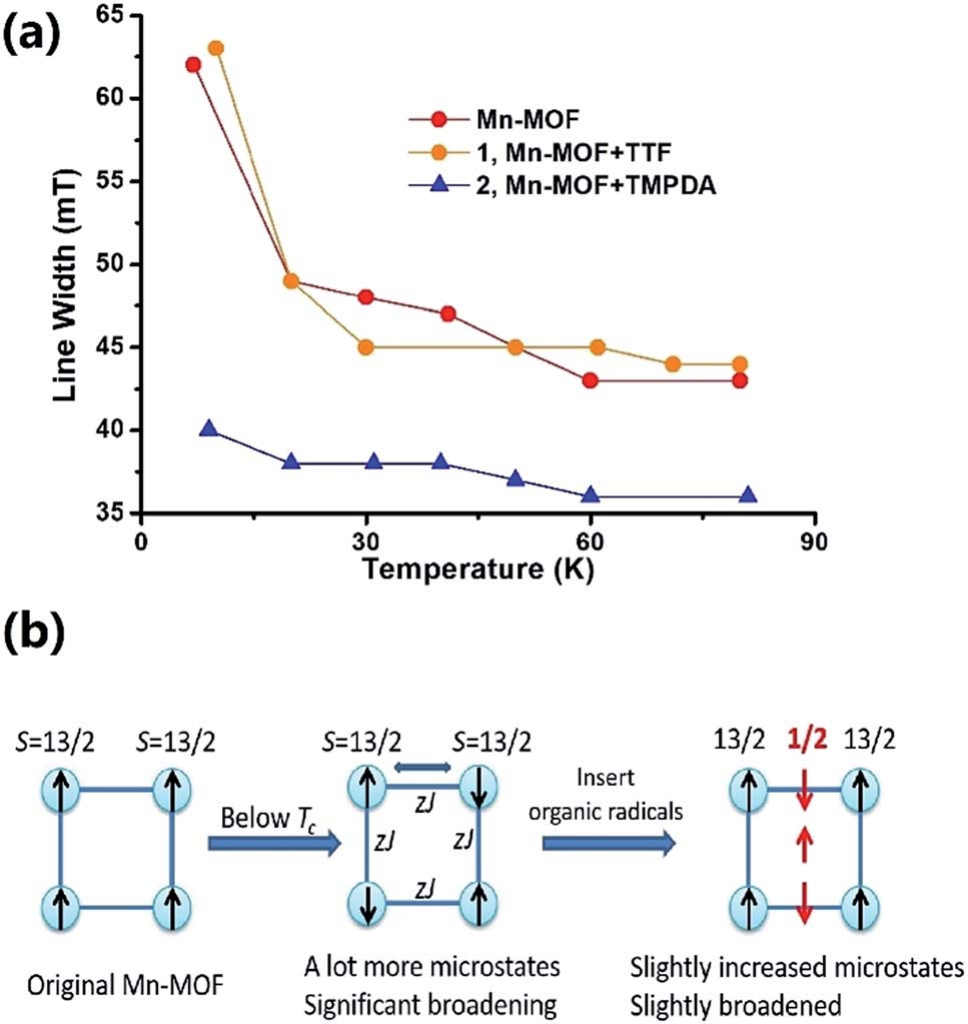
(a) Temperature-dependent EPR line-width change of Mn-MOF, **1** and **2**. (b) A possible scheme to explain the line width difference between Mn-MOF, **1** and **2**. This change in the line width indicated the existence of organic radical species in **2**.

The Weiss constants of Mn-MOF, **1** and **2** were fitted to be –15.0 K, –15.5 K and –21.4 K, respectively. For Mn-MOF and **1**, it was suggested that there were antiferromagnetic interactions between the SBUs. Since the skeleton of the framework in all three compounds is nearly identical, a slightly increased critical temperature of **2** might be a sign of the additional antiferromagnetic interactions between the SBUs and organic radicals. We confirmed this assumption with temperature variable EPR measurements (Fig. S11†). The EPR spectrum of the original Mn-MOF was first collected at a temperature range of 5–80 K. A wide peak appeared between 120 and 500 mT with *g* = 2.03, which corresponded to the numerous excited states and zero-field splitting feature of the *S* = 13/2 ground state of the Mn_7_ SBU. Moreover, at a temperature below the critical temperature (<10 K), the line width was notably broadened, suggesting that an increased population of excited states was generated by the interaction between the SBUs. A similar feature was also noticed for **1**, since the TTF species were almost neutral and contributed no perturbation to the spin states of the Mn_7_ cluster. In contrast, although the *g* value was not changed, the line width of **2** was slightly narrower at high temperature, and only slightly broadened when the temperature was lower than the *T*_c_ (Fig. 4a). Compared to those of Mn-MOF and **1**, the line width broadening of **2** below the critical temperature was miniscule. This phenomenon could be easily explained by the existence of organic radical spins. Since the organic radicals were placed between the Mn_7_ SBUs, the mean inter-SBU interactions were replaced by the interactions between the SBUs (*S* = 13/2) and organic radicals (*S* = 1/2). Hence, the number of microstates generated by the interactions was dramatically reduced (Fig. 4b).

### The confinement effect of the micropores enhanced the charge transfer phenomena

**Fig. 5:**
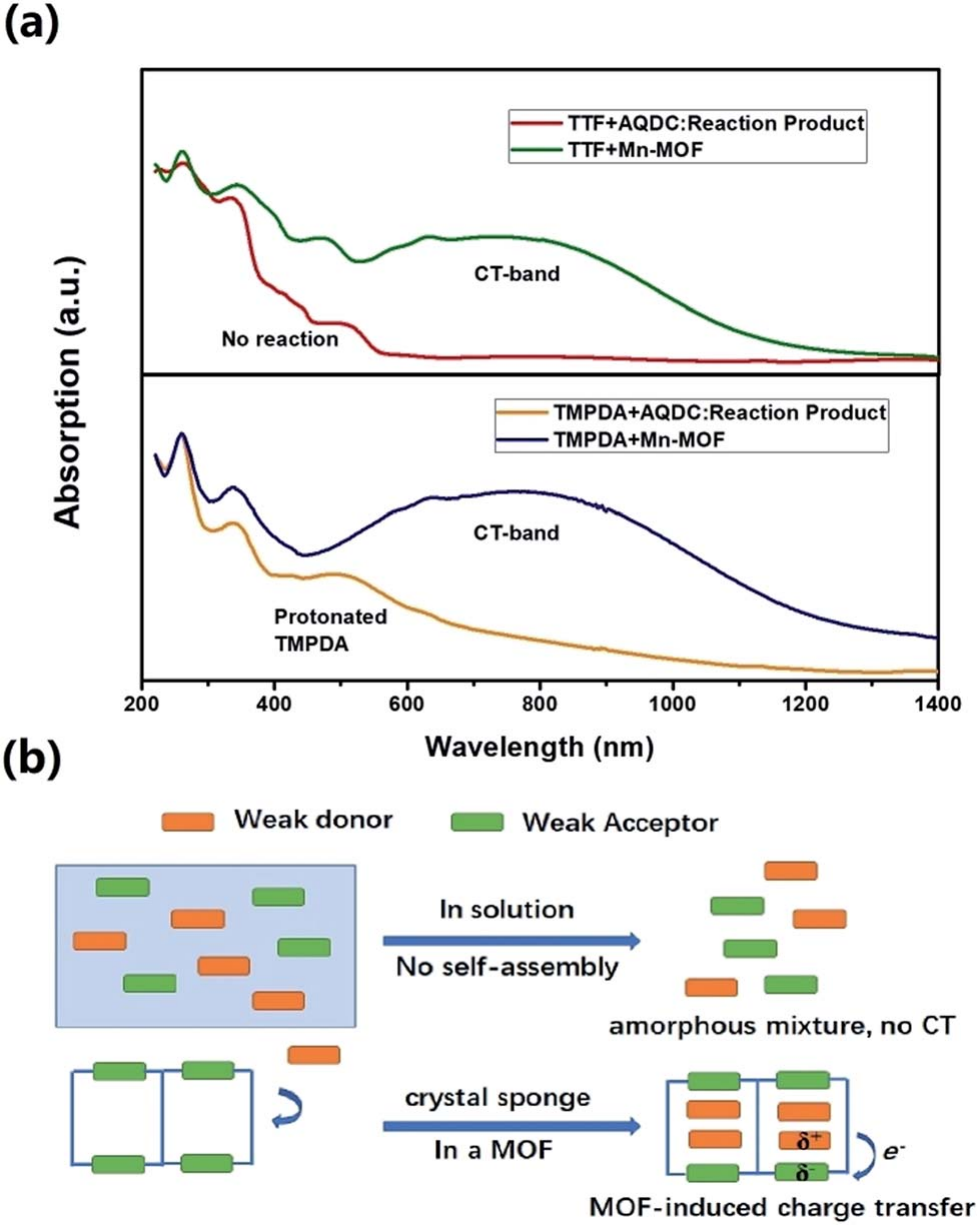
(a) Comparison between the solid-state UV-vis-NIR reflectance spectra of the guest impregnated Mn-MOF and that of the reaction product of the donor and AQDC ligand. In the case of TTF, the reaction product of TTF and AQDC showed no spontaneous reaction. In the case of TMPDA, the absorption band between 400 and 600 nm indicates a partial protonation of TMPDA, since the AQDC ligand is a carboxylic acid. (b) A scheme to illustrate the difference between the two conditions and the charge transfer induced by the MOF.

The cyclic voltammetry experiments of the AQDC ligand, TTF and TMPDA (Fig. S2†) unearthed an interesting fact: judging from the 1^st^ reduction potential of AQDC (*E*_1/2_ = –0.67 V *vs. E*_Ag/AgCl_) and the 1^st^ oxidation potential of TTF and TMPDA (*E*_1/2_ = 0.45 V and 0.37 V *vs. E*_Ag/AgCl_), the potential differences between the donor and acceptors were larger than 1.0 V in both cases. According to empirical rules, the combination of these donor molecules and AQDC molecules will not lead to a spontaneous formation of charge transfer salts but rather neutral complexes.^45^ This empirical assumption was proven with a simple reaction: a mixture of AQDC ligand and electron donor species was heated in DMF for 1 hour, followed by the evaporation of DMF, and the reaction residue was analysed with solid-state UV-Vis diffusive reflectance spectroscopy. Intriguingly, the absorption bands around 750 nm were absent for both TTF and TMPDA donor molecules (Fig. 5a). Unlike the dark green or dark blue colour of the Mn-MOF–guest species (Fig. S1†), both reaction residues exhibited a reddish-brown colour, which was the original colour of the donor molecules. Consequently, it can be stated that no charge transfer complexes can be simultaneously formed upon a reaction between the donor molecules and AQDC ligands. But in the case of a MOF, by evenly distributing these donor molecules in the micropores or channels in the scaffold, a “solid solution” state can be reached in which the donor and acceptor species are homogeneously mixed with respect to the molecular level. Moreover, the donor and acceptor species were confined to be at a very short distance; this confinement drastically promoted the charge transfer degree even between a weak donor and a weak acceptor (Fig. 5b). Consequently, by incorporating donors or acceptors into the skeleton of nanoporous MOF materials and taking up the counterparts, we were able to achieve unprecedented D–A charge transfer complexes that are inaccessible by conventional self-assembly methods.

## Conclusions

By post-synthetically inserting donor molecules into a flexible MOF with electron-accepting groups, we successfully achieved two MOF guest charge transfer complexes, and the structures of these complexes could be fully resolved using the “crystal sponge” approach. The nanopores or nanochannels in this MOF played a critical role in this process. The confinement of functional species in a limited space facilitated the guest-to-host electron transfer and yielded ionic charge transfer complexes, even when the redox potential gap between the donor and acceptor species was vast. Meanwhile, an even distribution of the donor molecules in the MOF structure maximized the contact between the donor and acceptor species, which also boosted the degree of charge transfer. The even distribution and confinement could be synergistically achieved in one MOF material, and this synergic effect can be summarized as “MOF-induced charge transfer” (see computational discussion in Section S11, ESI†). This strategy may lead to a new category of charge transfer complex, and may illustrate novel methods of modulating the electronic and magnetic properties of MOF materials. Indeed, in this manuscript, all reported charge transfer complexes were insulators (see the Discussion in ESI, S7†), but this fact was consistent with the structural analysis. By mediating the structure and topology of host MOFs, packing motifs that are favoured by semiconductors and metallic conductors, such as separated donor and acceptor columns, may also be realized.^46^ An appropriate packing motif would result in high conductivity and interesting physical properties, such as a superconductive phase transition like that observed in TTF–TCNQ. To sum up, this strategy is expected to lead to the discovery of various new materials, and could considerably enrich the application of MOFs in electronic and magnetic devices.

## Conflicts of interest

There are no conflicts to declare.

## Supplementary Material

Supplementary information

Crystal structure data
