# Supplementary material for: Nanopore-induced host–guest charge transfer phenomena in a metal–organic framework
Source: Chem Sci. 2018 Feb 15;9(13):3282–9. doi: 10.1039/c7sc05390h (PMC5916106; doi:10.1039/c7sc05390h)
Supplement: Supplementary file 1 [file SC-009-C7SC05390H-s001.pdf]

Electronic Supplementary Material (ESI) for Chemical Science.
This journal is © The Royal Society of Chemistry 2018

Supplementary Information.

Nanopore-induced Host-guest Charge Transfer Phenomena in a Metal-Organic Framework

S. Yamamoto, J. Pirillo, Y. Hijikata, Z. Zhang* and K. Awaga*
Department of Chemistry, Nagoya University, Furo-cho, Chikusa-ku, Nagoya 464-8602, Japan.
Institute of Transformative Bio-Molecules (WPI-ITbM), Nagoya University, Furo-cho, Chikusa-ku, Nagoya 464-8602, Japan.
Integrated Research Consortium on Chemical Sciences (IRCCS), Nagoya University, Furo-cho, Chikusa-ku, Nagoya 464-8602, Japan.

Table of Contents

S1. Pertinent Crystallographical Data.
S2. Optical image of Mn-MOF, **1** and **2**.
S3. Cyclic voltammetry of 2,7-AQDC, TTF and TMPDA and Mn-MOF.
S4. Additional structural details of **2**: bond angles.
S5. Powder X-ray diffraction (PXRD) pattern, simulated PXRD pattern and elemental analysis of **1** and **2** for verifying the purity.
S6. Selective impregnation of donor molecules into the Mn-MOF.
S7. Discussions about the electrical conductivity of **1** and **2**.
S8. Analysis of the magnetic properties of Mn-MOF, **1** and **2**.
S9. Temperature variable EPR signal of Mn-MOF, **1** and **2**.
S10. Raman spectrum of Mn-MOF, TTF and compound **1**.
S11. Theoretical simulations of molecular orbitals and absorption spectrum in **1** and **2**.

S1. Pertinent Crystallographical Data

Table S1. Pertinent crystallographic parameters and data for compounds **1** and **2**.

Compound	1 (CCDC: 1812408)	2 (CCDC: 1812409)
Formula	$C_{156}H_{116}Mn_7N_6O_{48}S_{20}$	$C_{200}H_{35}Mn_7N_{20}O_{48}$
F_w [g mol^{-1}]	3868.41	3905.42
Crystal size [mm^3]	0.15 x 0.07 x 0.06	0.07 x 0.05 x 0.04
Crystal system	Triclinic	Triclinic
Space group	P-1	P-1
a [Å]	14.3800	14.6390
b [Å]	15.3958	16.7430
c [Å]	21.7726	20.5510
α [°]	74.250	100.9130
β [°]	71.751	106.7800
γ [°]	82.351	100.1190
V [Å^3]	4399.85	4591.03
Z	1*	1*
ρ_{calc} [g cm^{-3}]	1.5051	1.4388
μ (Mo Kα) [mm^{-1}]	0.71075	0.75000 (Synchrotron)
Reflections collected	33309	88344
Unique reflections	18183	23143
Reflections with I >2σ(I)	12173	16467
Parameters	1114	1215
R(int)	0.0367	0.0387
*R*1[a]	0.0801	0.0883
*wR*2[b]	0.2057	0.2574
GOF	1.062	1.064

[a] $R1 = \Sigma||F_o| - |F_c||\Sigma|F_o|$. [b] $wR2 = [\Sigma[w(F_o^2 - F_c^2)^2]/\Sigma[w(F_o^2)^2]]^{1/2}$.

*: In cif files, since the space group is P-1, so Z=2. Therefore, to make the data consistent, the formula in cif files were divided by 2. In this table, the formula was based on an individual Mn_7 cluster, and

therefore the Z was set to 1.

S2. Optical image of Mn-MOF, 1 and 2.

(a)

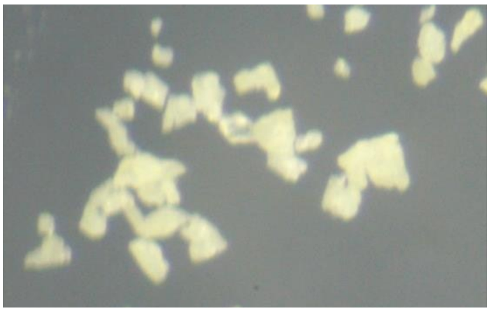

(b)

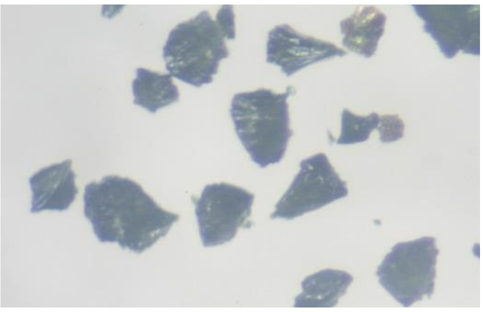

(c)

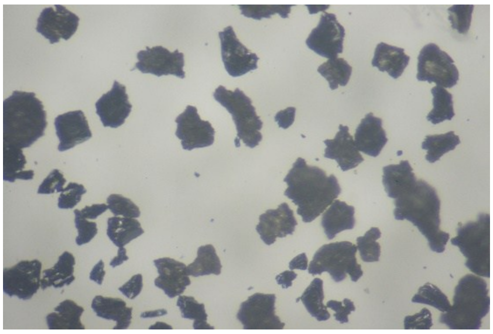

Fig. S1 Optical image of (a) Mn-MOF, (b) **1** and (c) **2**.

S3. Cyclic voltammetry of 2,7-AQDC ligand, TTF and TMPDA

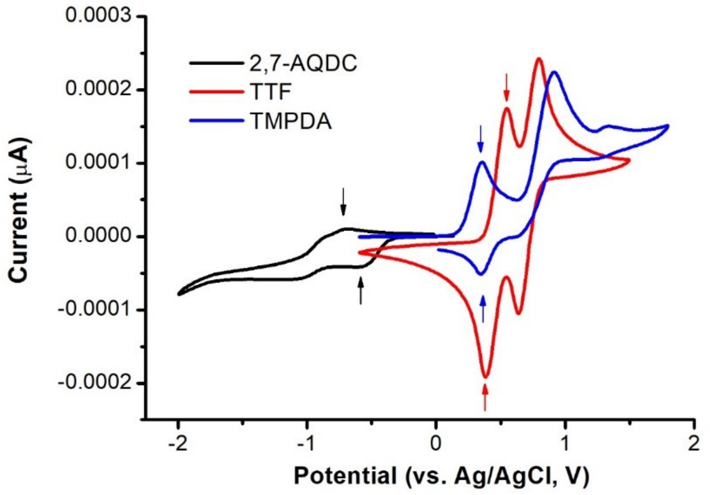

Fig. S2 Cyclic voltammetry of 2,7-AQDC, TTF and TMPDA. $E_{1/2}$ of the first reduction event of 2.7-AQDC was -0.67V, while $E_{1/2}$ of the first oxidation event of TTF and TMPDA were 0.45V and 0.37V respectively. The measurements were performed with a saturated DMF solution, with 1.0 mol/L $[Bu_4N]PF_6$ as the electrolyte.

Note: Since the TTF and TMPDA molecules will be released from MOF while immersing the crystals of **1** and **2** in organic solvents, it is meaningless to perform solid-state CV on them. The electrolyte will decompose **1** and **2**, leaving a mixture of Mn-MOF and donor molecules.

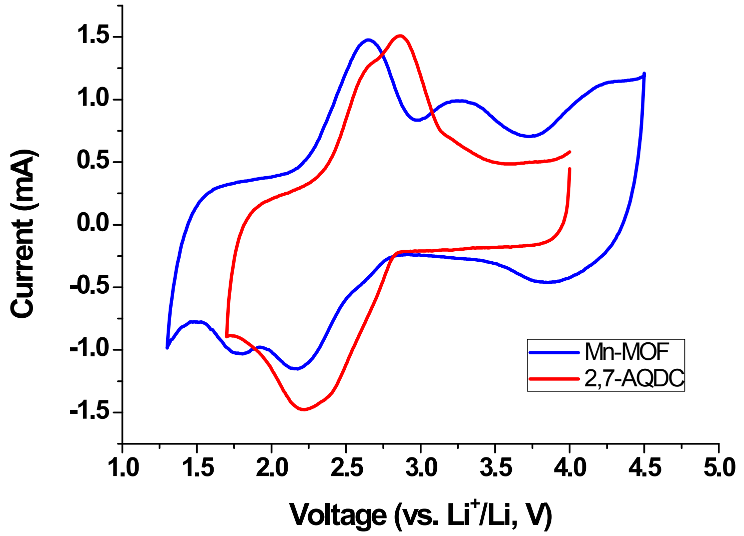

Fig. S3 Solid-state cyclic voltammetry of Mn-MOF and 2,7-AQDC, the measurement condition is the same as described in an previously published article by us. [S1] In both cases, $E_{1/2}$ of anthraquinone moiety is around 2.4-2.5V. In Mn-MOF, the reduction and oxidation events are further separated, indicating a strong kinetic effect that is caused by the poor conductivity of MOF material. However, the thermodynamic reduction potential of AQDC is barely modified in the Mn-MOF.

S4. Additional structural details of **2**: bond angles.

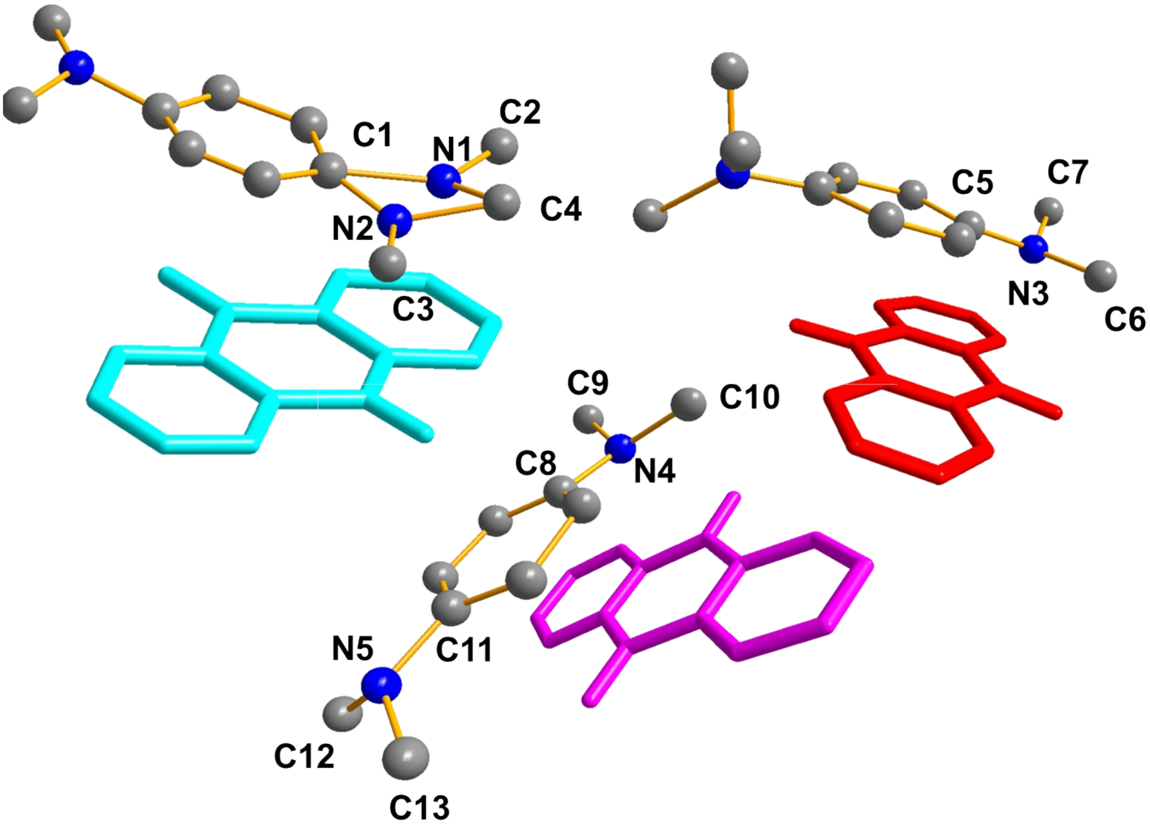

Fig. S4 In the main article, we pointed out that in compound **2**, some N atoms in TMPDA molecules were perturbated from the original sp^2 hybridization and possessed some sp^3 character. Herein we used some selected bond angles to explain this observation. Bond angles are listed in **Table S2**.

Table S2 Selected bond angles. Bond angles are listed in degree.

∠C1-N1-C2	137.9	∠C1-N1-C4	115.4
∠C2-N1-C4	102.1	Average	118.5
∠C1-N2-C3	121.1	∠C1-N2-C4	127.1
∠C3-N2-C4	98.7	Average	115.6

∠C5-N3-C7	114.3	∠C5-N3-C6	118.8
∠C6-N3-C7	116.6	Average	116.6

∠C8-N4-C9	111.5	∠C8-N4-C10	118.6
∠C9-N4-C10	118.5	Average	116.0
∠C11-N5-C12	115.6	∠C11-N5-C13	117.8
∠C12-N5-C13	117.0	Average	116.8

As listed in Fig. S3 and Table S2, these methyl groups are not coplanar with the C-N bonds that connect the dimethylamino group and phenyl rings. Bond angles are also slightly deviated from 120º, indicating a partial sp^3 character of N atoms.

S5. Powder X-ray diffraction (PXRD) patterns and simulated PXRD patterns of **1** and **2**.

Firstly, to verify the purity of **1** and **2**, PXRD patterns were collected for **1** and **2**, and the diffraction patterns were compared to their structure simulated patterns.

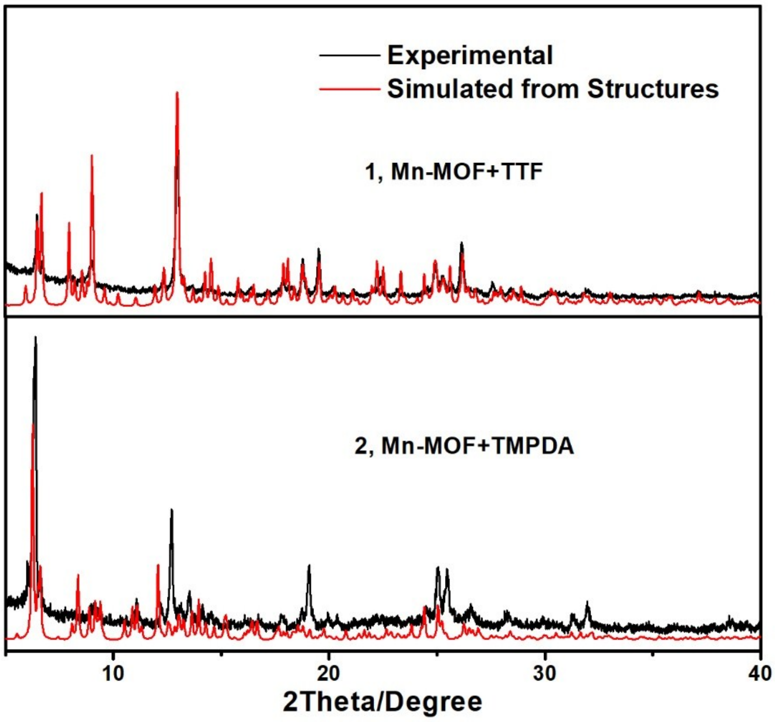

Fig. S5 The experimental diffraction patterns (black) and pattern simulated from the structure (red) of **1** and **2**.

Furthermore, we also performed the CHN elemental analysis on a collection of crystals of **1** and **2**, using a YANACO MT-6 elemental analyzer. Compound **1** ($Mn_7C_{166}H_{116}O_{48}N_6S_{20}$): Calc: C: 49.99 H: 2.93 N: 2.11 (%) Found: C: 49.18 H: 2.96 N: 1.46 (%). Compound **2** ($Mn_7C_{198}H_{194}O_{48}N_{18}$): Calc: C: 59.28 H: 4.97 N: 6.41 (%) Found: C: 58.55 H: 4.74 N: 5.88 (%).

Combining the result of PXRD and elemental analysis, the bulk sample of **1** and **2** could be considered as phase-pure samples.

S6. Selective impregnation of donor molecules into the Mn-MOF.

We have also attempted the spontaneous incorporation of BEDT-TTF and TMTSF into the MOF crystals. (Fig. S5) However, since the pore size is limited (~1nm) and the size of these donor molecules are quite big, the diffusion of these molecules in nanochannels are very slow and only the surface of crystals could be doped with these donors. Therefore, no phase pure materials could be isolated. For the evidences, see the result of SEM-EDX analysis. (Fig. S6, collected on a JEOL JES-FA200 scanning electron microscope with energy dispersive X-ray spectroscopy setup)

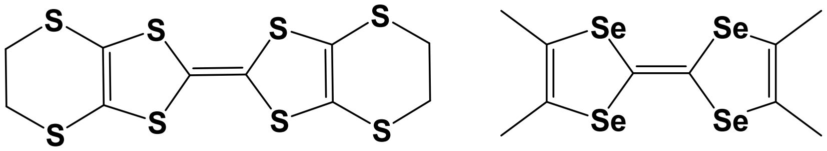

BEDT-TTF **TMPDA**

Fig. S6 Molecule structure of bis(ethylenedithio)tetrathiafulvalene (BEDT-TTF) and 4,4',5,5'-tetramethyl-Δ2,2'-bi(1,3-diselenole) (TMTSF).

(a)

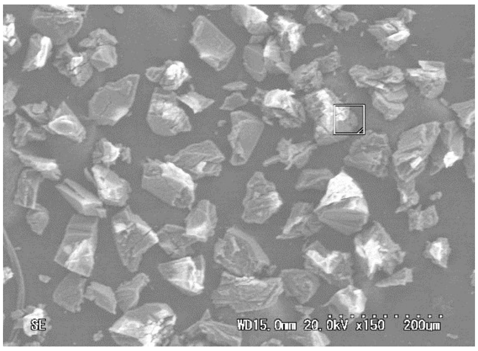

(b)

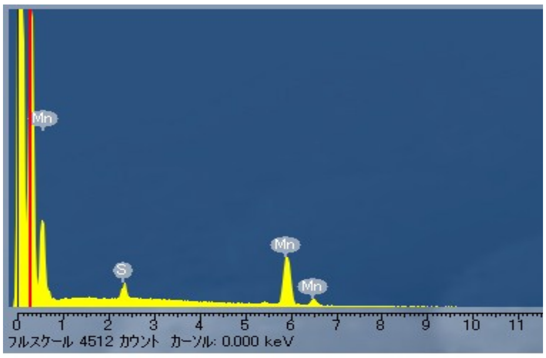

(c)

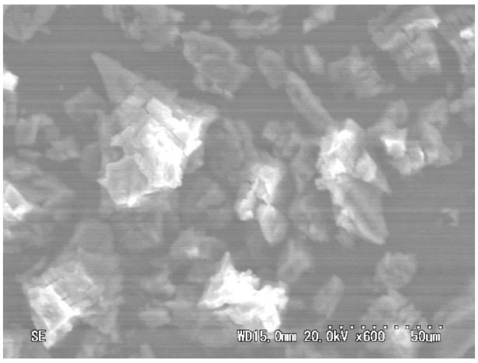

(d)

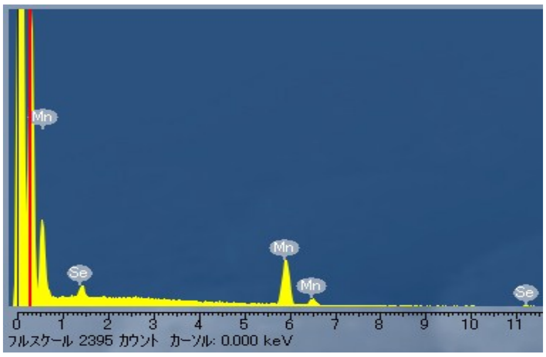

Fig. S7 SEM image **(a,c)** and EDX spectrum **(b,d)** of BEDT-TTF and TMTSF doped Mn-MOF, respectively. The amount of doped BEDT-TTF could be estimated by the molar ratio of sulfur, and the amount of TMTSF could be estimated by the molar ratio of selenium. See Table S3.

Table S3. Molar ratio of sulfur and selenium in BEDT-TTF and TMTSF doped Mn-MOF. For convenient, only Mn, S and Se elements were counted in the analysis.

BEDT-TTF doped MOF:

	Molar% of S	Molar% of Mn	S: Mn
Area 1	18.93	81.07	1: 4.283
Area 2	27.97	72.03	1:2.575
Area 3	13.17	86.83	1:6.593
Average S/Mn ratio	1:4.483		

Since BEDT-TTF molecule contains 8 sulfur atoms, the formula could be estimated as $Mn_7(AQDC)_7(DMA)_6$□ BEDT-$TTF_{0.20}$.

TMTSF doped MOF:

	Molar% of Se	Molar% of Mn	Se: Mn
Area 1	17.28	82.72	1: 4.787
Area 2	15.17	84.83	1:5.592
Area 3	16.34	83.66	1:5.120
Average Se/Mn ratio	1:5.167		

Since TMTSF molecule contains 4 selenium atoms, the formula could be estimated as $Mn_7(AQDC)_7(DMA)_6$□ $TMTSF_{0.34}$.

Due to the limited amount of donor molecules, we couldn’t get a phase pure charge transfer complex in either case. Apparently, this strategy for preparing MOF□ guest charge transfer complexes are highly selective. The restrictions arise from the pore size of MOFs and the vibrational diameter of inserted organic molecules.

S7. Discussions about the electrical conductivity of **1** and **2**.

After the incorporation of donor molecules, due to the crack of crystals, it is difficult to find a crystal with a regular and rectangular shape. Therefore, we used 2-probe method to characterize the electrical conductivity of pressed pellets of **1** and **2** with an Advantest R6245 multimeter. Placing samples under vacuum, we can't detect the current signal through the pressed pellet of **1** and **2** even with an applied voltage of 50V, suggesting the conductivity of these materials are lower than 10^{-12} S/cm.

Interestingly, upon the exposure to a 97% RH (relative humidity), we observed a linear I-V curve for **1** and a I-V curve with hysteresis for **2** (**Fig. S6**). The hysteresis in the I-V characteristics for compound **2** might be induced by the hopping of protons in the material. However, after 24-hour exposure to a 97% RH, both **1** and **2** were decomposed and lose crystallinity. Hence, we don't think this humidity induced conductivity is an intrinsic property of **1** and **2**. Similar feature has been observed in previous reports. [S1]

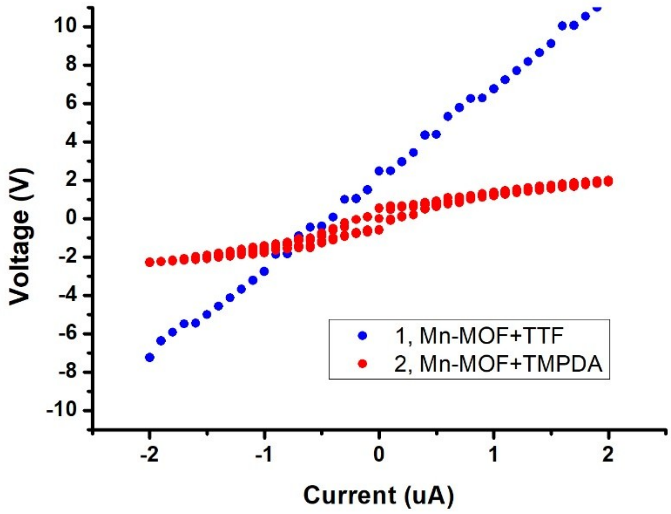

Fig. S8 I-V characteristics of **1** and **2**. The measurement was performed with a variable current method, and the range of current is -2 μA to 2 μA.

S8. Analysis of the magnetic properties of Mn-MOF, **1** and **2**.

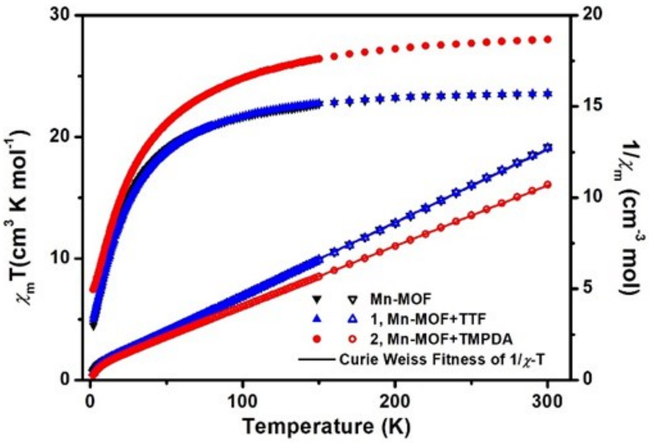

Fig. S9 χT-T and 1/χ-T plot of Mn-MOF, compound **1** and **2**. The data sets between 50-300K were fitted with Curie-Weiss law. The Curie constant and critical temperature was 24.84 $cm^3 \cdot mol^{-1} \cdot K$ and -15.0K for Mn-MOF, 24.95 $cm^3 \cdot mol^{-1} \cdot K$ and -15.5K for **1**, and 30.46 $cm^3 \cdot mol^{-1} \cdot K$ and -21.4K for **2**.

Curie-weiss Fitness: Using least square approach, we fitted the 1/χ -T plot of Mn-MOF, 1 and 2 to the Curie-weiss law:

$$\chi = C/\ (\mathrm{T}\text{-}\Theta) \qquad \text{Equation S1.}$$

And the *C*, Curie constant, will be used in the following equations to estimate the number of spins.

$$\mathrm{C} = \Sigma\ \{\mu_B{}^2 N g^2 S(\mathrm{S}+1)/3k_B\} \qquad \text{Equation S2.}$$

For Mn-MOF itself, the χT-T measurement suggested the Curie constant to be 24.84 $cm^3 \cdot mol^{-1} \cdot K$. Since the SBU (secondary building unit) is a heptanuclear cluster with 7 Mn(II) cations, the physical model could be simplified to a Mn_7 cluster with weak intermolecular interactions. However, it is too resource consuming for the software to fit the exchange coupling features in such a heptanuclear cluster with large spin numbers, we decided to treat the data with a gigantic spin approximation. The data fitness was performed with PHI,[S2] with isotropic *g*-factor and *zJ* being independent variables. (**Fig. S9**)

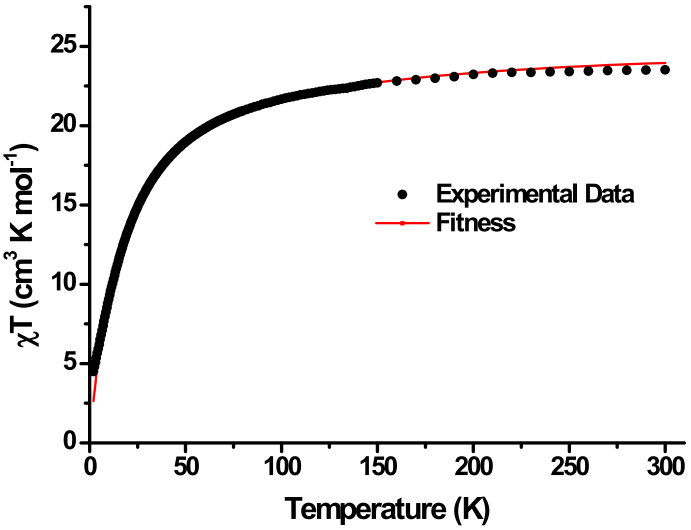

Fig. S10 χT-T data and fitness of Mn-MOF. The best fitness gives S=13/2, g=2.04 (experimental value from EPR: g=2.03), and zJ = -0.177cm^{-1}. Residue=0.11%.

The magnetic contribution of organic radicals was estimated by the following methods: By fit 1/χ -T plots to Equation S1, we can get C_1, C_2 and $C_{\text{Mn-MOF}}$. Let $C_{\text{TTF+AQ}}$ = C_1-$C_{\text{Mn-MOF}}$ and $C_{\text{TMPDA+AQ}}$ = C_2-$C_{\text{Mn-MOF}}$, and $N_{\text{TTF+AQ}}$ and $N_{\text{TMPDA+AQ}}$ could be calculated with Equation S2. Since the charge of donor molecules and acceptor molecules should be same, $N_{\text{TTF+AQ}}$ = 2N_{TTF}, and $N_{\text{TMPDA+AQ}}$ = 2N_{TMPDA}. Herein, we ignored the potential antiferromagnetic coupling of donor species and acceptor species, as well as the coupling between the Mn_7 clusters and organic radicals. Therefore, this result is a qualitative result.

S9. Temperature variable EPR signal of Mn-MOF, **1** and **2**.

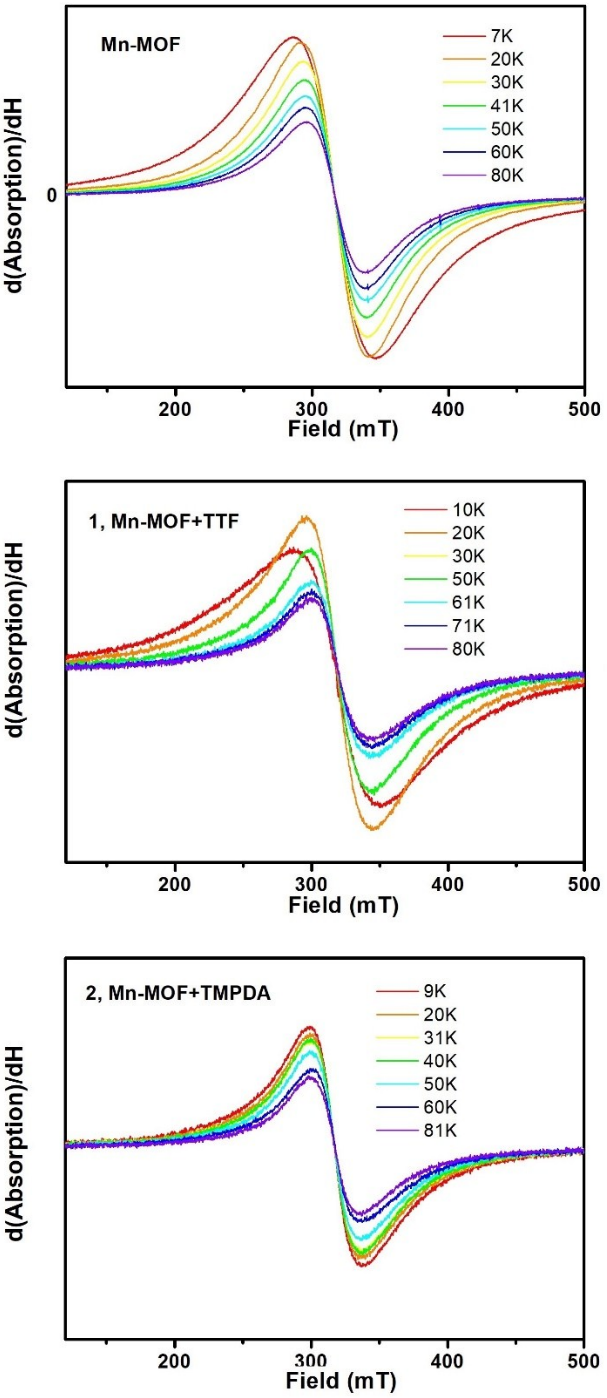

Fig. S11 Temperature variable EPR signal of Mn-MOF, **1** and **2**. Significant line width broadening could be observed for Mn-MOF and **1** when the measurement temperature is below T_c, but for **2**, the broadening under low temperature is not obvious.

S10. Raman spectrum of Mn-MOF, TTF and compound **1**.

The Raman spectrum of Mn-MOF, TTF and compound **1** was carried on a Photon Design RSM-310 Raman spectrometer with an excitation laser wavelength of 1064nm in Chitose institute of science and technology.

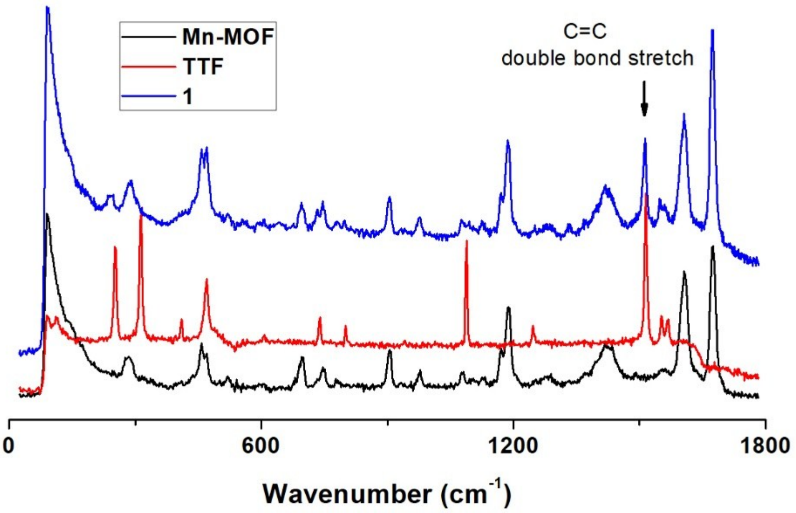

Fig. S12 Raman spectrum of Mn-MOF, TTF and compound **1**. The trademark C=C double bond stretch appears at 1514cm^{-1} for TTF molecule and appears at 1512cm^{-1} for compound **1**. Although a peak shift to lower wavenumber indicates a single bond component arise from the radical species, the peak shift in this case is barely noticeable and suggest the charge transfer degree in **1** is extremely small.

S11. Theoretical simulations of molecular orbitals and absorption spectrum in **1** and **2**.

Computational details: In order to investigate the origin of the broad peaks in UV-Vis-NIR spectra of **1** and **2**, we performed density functional theoretical (DFT) calculations using Gaussian 09. [S4] We constructed dimer (D_{1a} and D_{2a} for **1**; D_{1b}, D_{2b}, and D_{3b} for **2**) and tetramer (T_{1a} for **1**; T_{1b}, T_{2b}, and T_{3b} for **2**) models referring to the crystal structures. As shown in Figure S12, we kept the geometries and alignments of the guest molecules and AQDC in the crystal structures, except for H atoms and highly disordered atoms which were theoretically optimized, followed by time-dependent DFT (TD-DFT)[S5] calculation to simulate UV/Vis spectra. All optimizing calculations were carried out at the M06/6-311G** level of theory, [S6] and all TD-DFT calculations were performed at the M06/6-311+G* level of theory.

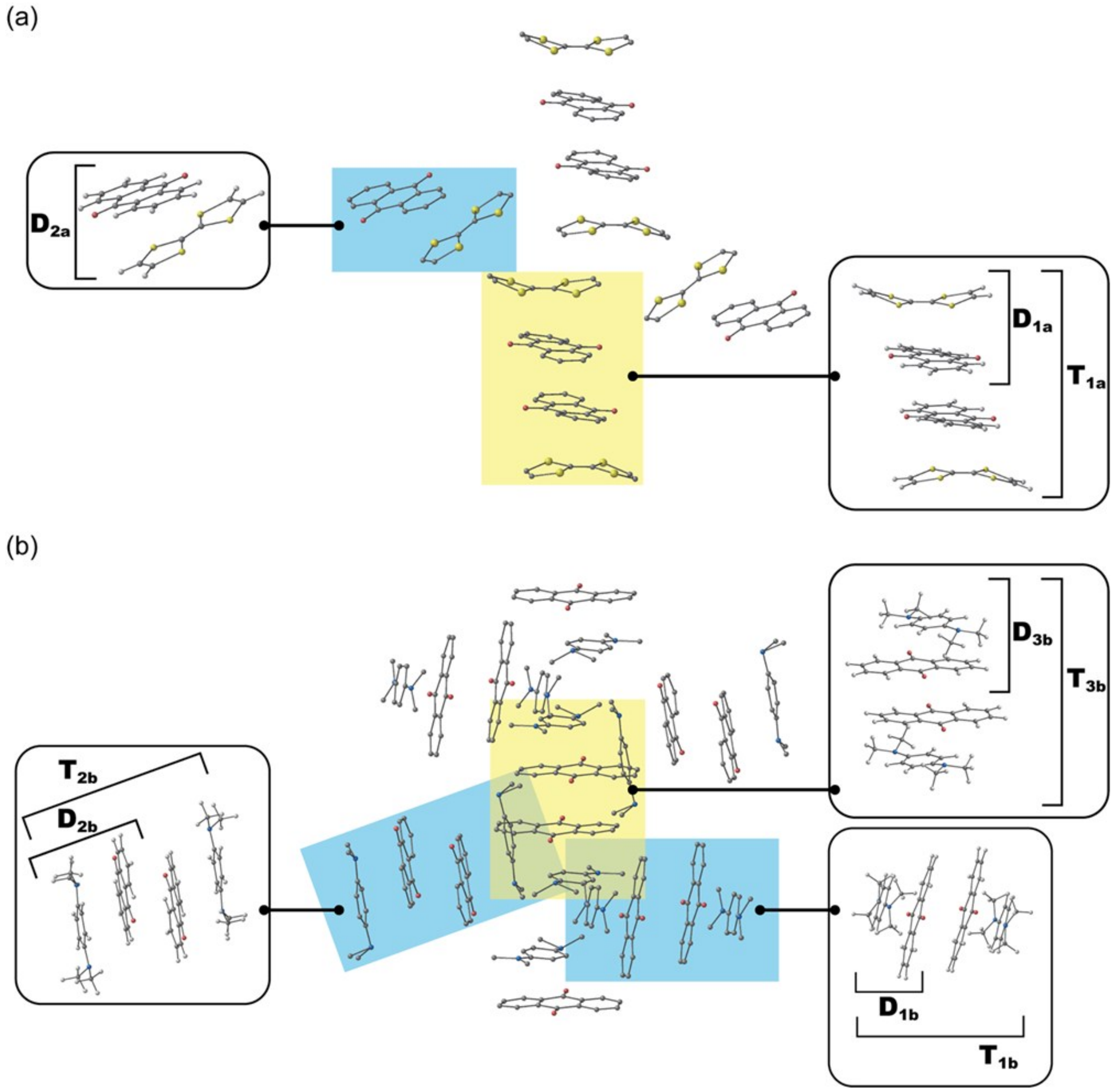

Figure S13. Model structures for (a) 1 (D_{1a}, D_{2a}, and T_{1a}) and (b) for 2 (D_{1b}, D_{2b}, D_{3b}, T_{1b}, T_{2b}, and T_{3b}). The crystal structures of guest and ligand alignments were shown in the center of each figure, where yellow and blue colors indicate the employed models. Gray, red, blue, yellow, and white indicate carbon, oxygen, nitrogen, sulfur, hydrogen atoms, respectively..

Molecular orbitals and corresponding CT bands.

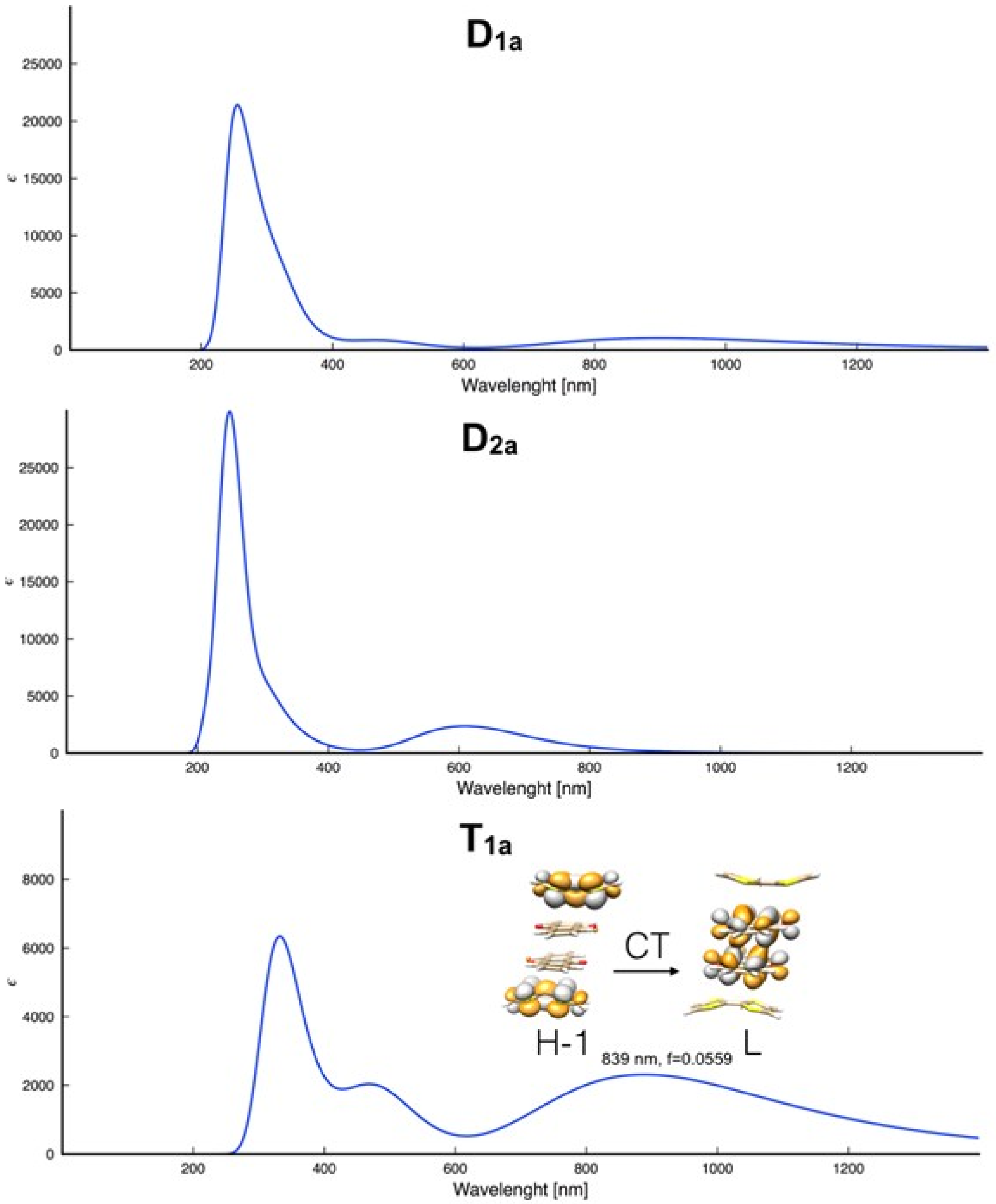

Figure S14. Simulated absorption spectra of D-A dimers (D_{1a}, D_{2a}) and D-A-A-D tetramers (T_{1a}) in the structure of compound **1**. Apparently, the broad charge transfer band that was observed in the experiment was induced by the charge transfer phenomena within D-A-A-D tetramers. In reality, the D-A-A-D tetramers are connected into columns, which may result a broader electronic absorption peak as shown in the experimental UV-Vis-NIR spectrum.

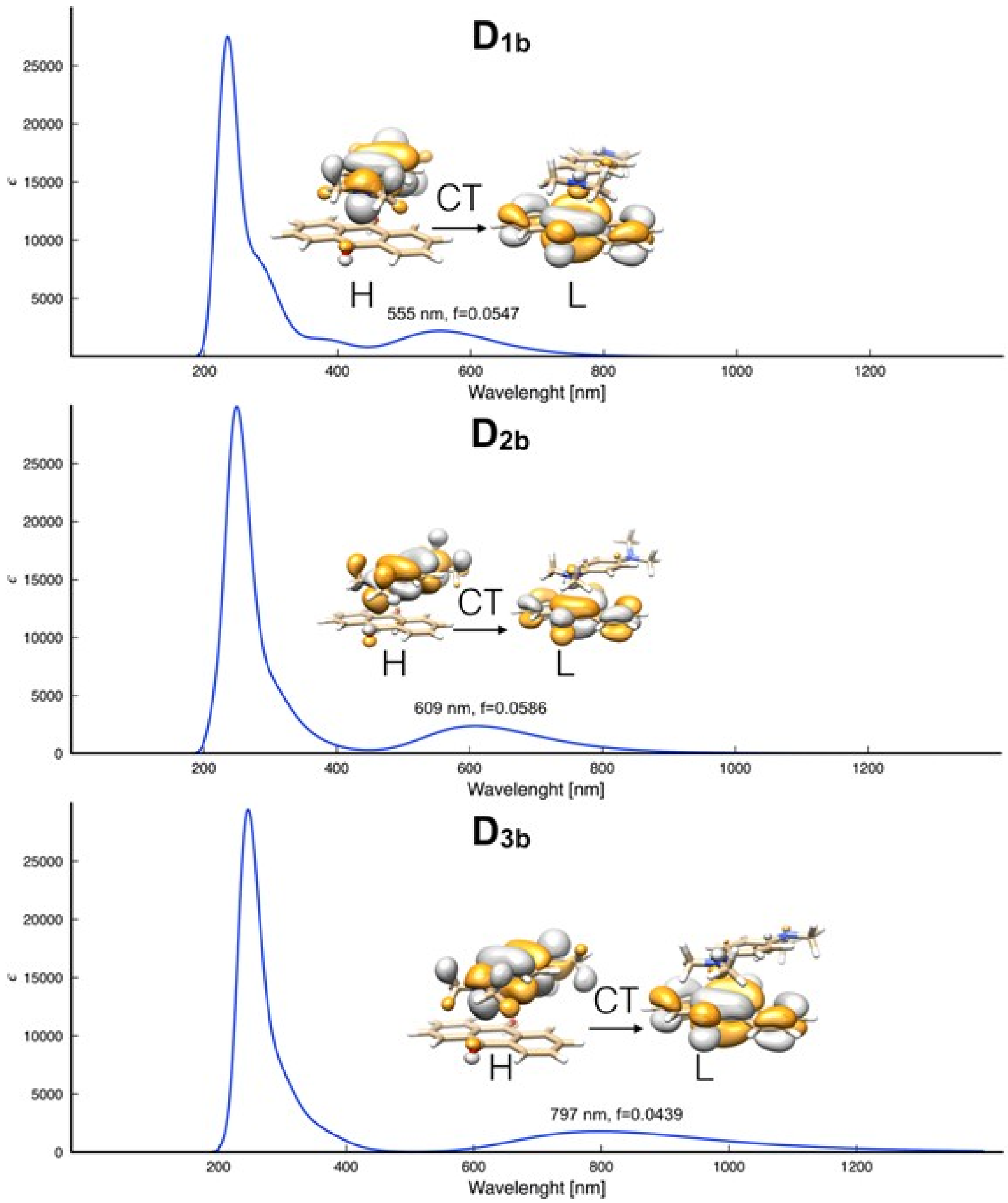

Figure S15. Simulated absorption spectra of D-A dimers (D_{1b}, D_{2b} and D_{3b}) in the structure of compound 2. For all three D-A dimers, the charge transfer from HOMO to LUMO bands around 600-800nm are very weak.

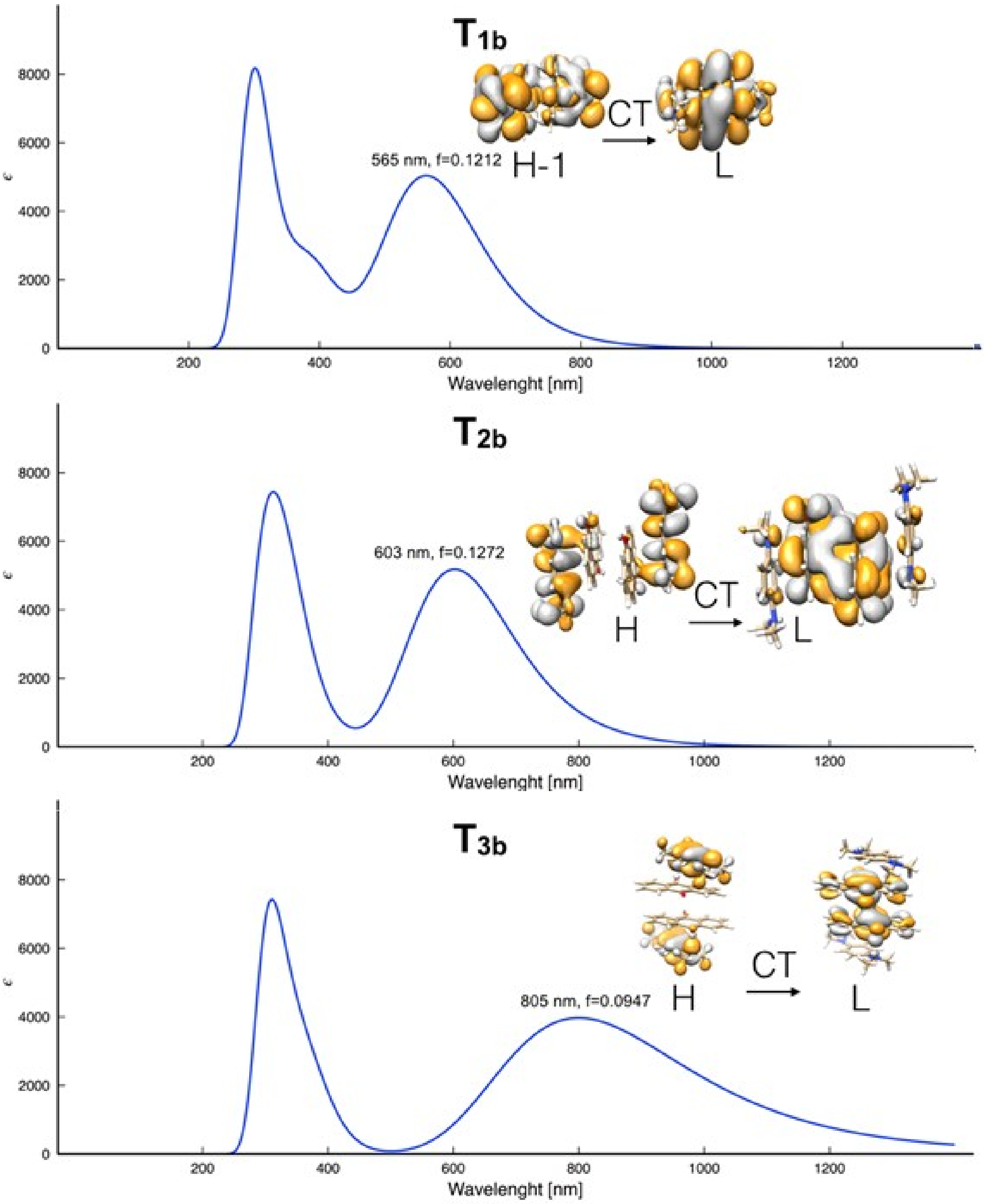

Figure S16. Simulated absorption spectra of D-A-A-D tetramers (T_{1b}, T_{2b} and T_{3b}) in the structure of compound **2**. Apparently, the formation of tetramer significantly enhanced the accessibility of charge transfer phenomena and lead to several absorption peaks at longer wavelength (in other words, lower energy).

Fig. S14-S16 clearly illustrated that the absorption around 700-800nm in the experiment arises from the charge transfer phenomena between the donor guest molecules and host frameworks. To be more specific, the charge transfer phenomena within a D-A-A-D tetrameric unit is mainly responsible for the absorption bands that have been observed in UV-Vis-NIR.

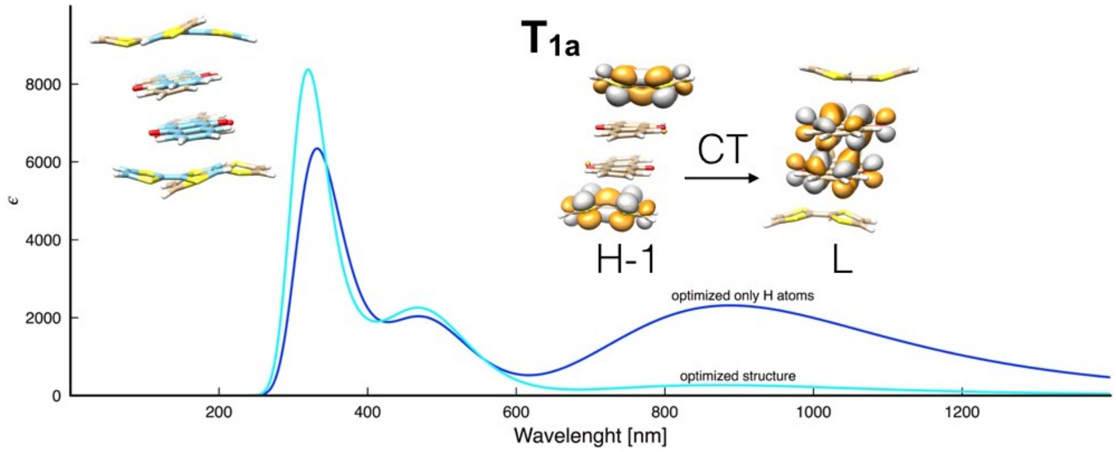

Figure S17. Simulated absorption spectrum of the original D-A-A-D tetramer from the structure of 1, and the tetramer after the geometry optimization. After the optimization, the bending directions of TTF molecules have been reversed, and the absorption peak at ~900nm disappeared. Apparently, the original structure of D-A-A-D tetramer, which is fixed by the steric effect of nanopores, is providing a low energy pathway to the charge transfer phenomena.

Conclusively, these computational results suggested two effects that enhance the CT phenomena: 1, the formation of D-A-A-D tetramer. 2, the steric effect of nanopores. Since both effects come from the specific structure of the Mn-MOF, it can be recognized as “MOF-induced charge transfer”.

In order to obtain more insights into effects of the AQDC coordination to the Mn ions on the molecular orbitals (MOs) of AQDC, we compared the MOs of free AQDC, AQDC dimer and Mn-AQDC models in the same geometry as the crystal structure, as shown in Figure S17. The AQDC dimer and the Mn-AQDC were terminated by H atoms which were optimized. We optimized the AQDC dimer in singlet state and the Mn-AQDC in the highest spin state at the 6-311G**/M06 level of theory using Gaussian 09.

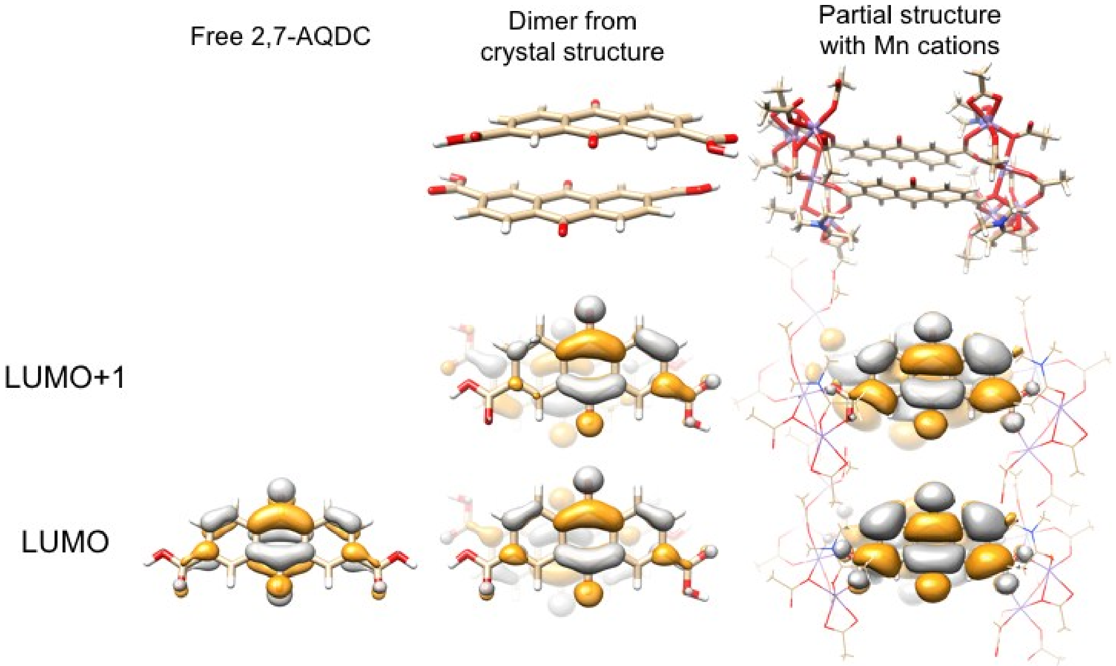

Figure S18. Frontier orbital of a free 2,7-AQDC ligand, 2,7-AQDC dimer from the structure, and a partial structure of Mn-MOF containing a 2,7-AQDC pair and the Mn(II) cations that connect to the 2,7-AQDC pair. In the case of dimer and dimer with Mn(II) cations, the LUMO and LUMO+1 are degenerate as two AQDC moieties in the system are crystallographically equivalent. All 3 LUMOs are composed by the molecular orbital of AQDC ligand, and the symmetry of them is the same. Since this LUMO of AQDC is the orbital that accepts the outcoming electrons from organic donors and forms radical species, from the calculation result, the LUMO orbital of AQDC is not significantly perturbed by the coordination bonds between carboxylate groups and Mn(II) cations. This result agrees with the solid-state cyclic voltammetry in section S3.

References:

S1. Z. Zhang, H. Yoshikawa and K. Awaga, *Chem. Mater.*, 2016, **28**, 1298.

S2. L. Sun, S. S. Park, D. Sherbela and M. Dincă, *J. Am. Chem. Soc.,* 2016, **138**, 14772.

S3. N. F. Chilton, R. P. Anderson, L. D. Turner, A. Soncini and K. S. Murray, *J. Comput. Chem.* 2013, **34**, 1164.

S4. Gaussian 09, (Revision D.01), M. J. Frisch, G. W. Trucks, H. B. Schlegel, G. E. Scuseria, M. A. Robb, J. R. Cheeseman, G. Scalmani, V. Barone, B. Mennucci, G. A. Petersson, H. Nakatsuji, M. Caricato, X. Li, H. P. Hratchian, A. F. Izmaylov, J. Bloino, G. Zheng, J. L. Sonnenberg, M. Hada, M. Ehara, K. Toyota, R. Fukuda, J. Hasegawa, M. Ishida, T. Nakajima, Y. Honda, O. Kitao, H. Nakai, T. Vreven, J. A. Montgomery, Jr., J. E. Peralta, F. Ogliaro, M. Bearpark, J. J. Heyd, E. Brothers, K. N. Kudin, V. N. Staroverov, R. Kobayashi, J. Normand, K. Raghavachari, A. Rendell, J. C. Burant, S. S. Iyengar, J. Tomasi, M. Cossi, N. Rega, J. M. Millam, M. Klene, J. E. Knox, J. B. Cross, V. Bakken, C. Adamo, J. Jaramillo, R. Gomperts, R. E. Stratmann, O. Yazyev, A. J. Austin, R. Cammi, C. Pomelli, J. W. Ochterski, R. L. Martin, K. Morokuma, V. G. Zakrzewski, G. A. Voth, P. Salvador, J. J. Dannenberg, S. Dapprich, A. D. Daniels, Ö. Farkas, J. B. Foresman, J. V. Ortiz, J. Cioslowski, D. J. Fox, Gaussian, Inc., Wallingford CT, 2009.

S5. M. E. Casida, Recent Developments and Applications in Density-Functional Theory, (Eds: Seminario JM), Amsterdam, The Netherlands: Elsevier, 1996, 155.

S6. Y. Zhao and D. G. Truhlar, *Theor. Chem. Acc.* 2008, **120**, 215.
